# Quantification of experimental venous thrombus resolution by longitudinal nanogold-enhanced micro-computed tomography

**DOI:** 10.1016/j.thromres.2015.10.006

**Published:** 2015-12

**Authors:** Steven P. Grover, Prakash Saha, Julia Jenkins, Arun Mukkavilli, Oliver T. Lyons, Ashish S. Patel, Kavitha Sunassee, Bijan Modarai, Alberto Smith

**Affiliations:** a Academic Department of Vascular Surgery, Cardiovascular Division, King's College London, BHF Centre of Research Excellence & NIHR Biomedical Research Centre at King's Health Partners, St Thomas' Hospital, London, UK; b Division of Imaging Sciences and Biomedical Engineering, King's College London, Wellcome Trust — EPSRC Medical Engineering Centre & NIHR Biomedical Research Centre at King's Health Partners, St. Thomas' Hospital, London, UK

**Keywords:** Venous thrombosis, Animal models, Nanogold, MicroCT

## Abstract

**Introduction:**

The assessment of thrombus size following treatments directed at preventing thrombosis or enhancing its resolution has generally relied on physical or histological methods. This cross-sectional design imposes the need for increased numbers of animals for experiments. Micro-computed tomography (microCT) has been used to detect the presence of venous thrombus in experimental models but has yet to be used in a quantitative manner. In this study, we investigate the use of contrast-enhanced microCT for the longitudinal assessment of experimental venous thrombus resolution.

**Materials and methods:**

Thrombi induced by stenosis of the inferior vena cava in mice were imaged by contrast-enhanced microCT at 1, 7 and 14 days post-induction (n = 18). Thrombus volumes were determined longitudinally by segmentation and 3D volume reconstruction of microCT scans and by standard end-point histological analysis at day 14. An additional group of thrombi were analysed solely by histology at 1, 7 and 14 days post-induction (n = 15).

**Results:**

IVC resident thrombus was readily detectable by contrast-enhanced microCT. MicroCT-derived measurements of thrombus volume correlated well with time-matched histological analyses (ICC = 0.75, P < 0.01). Thrombus volumes measured by microCT were significantly greater than those derived from histological analysis (P < 0.001). Intra- and inter-observer analyses were highly correlated (ICC = 0.99 and 0.91 respectively, P < 0.0001). Further histological analysis revealed noticeable levels of contrast agent extravasation into the thrombus that was associated with the presence of neovascular channels, macrophages and intracellular iron deposits.

**Conclusion:**

Contrast-enhanced microCT represents a reliable and reproducible method for the longitudinal assessment of venous thrombus resolution providing powerful paired data.

## Highlights

• Contrast-enhanced microCT allows longitudinal measurements of experimental venous thrombi.
• Measurements of thrombus volume by microCT are highly reproducible.
• Aurovist nano-gold contrast accumulates in highly organised regions of the resolving thrombus.

## 1 Introduction

Experimental models enable the cellular and molecular mechanisms that regulate the formation and resolution of venous thrombi to be studied and have afforded considerable insight into the molecular and cellular mediators of these processes [1], [2], [3]. However, previous studies have relied largely on end-point analysis of thrombus weight or ‘size’, or a combination of histology and image analysis of sections taken through the thrombus. Advances in imaging techniques have facilitated the *in vivo* longitudinal measurement of thrombus, with magnetic resonance imaging (MRI) using a clinical 3 T scanner for determination of thrombus volume in the experimental setting being recently reported [4], [5]. MRI accurately quantifies thrombus size in murine models of venous thrombosis with high resolution, but access to these scanners is limited because of the cost of this imaging modality. High frequency ultrasound has also been used to study experimental models of venous thrombosis, but although affordable requires a high degree of technical skill and so far only provides two-dimensional imaging of the thrombus [6], [7].

Contrast-enhanced computed tomography (CT) has been used to demonstrate the presence of thrombus in man. Technological advances in the field of CT have facilitated the development of high-resolution micro-computed tomography (microCT) imaging platforms suitable for pre-clinical use [8]. The availability of microCT facilities has increased considerably in the past decade and represents an affordable and readily accessible methodology for pre-clinical imaging. MicroCT has been used extensively in the study of murine models of cardiovascular pathologies including critical limb ischaemia, abdominal aortic aneurysm and myocardial infarction [9], [10], [11]. Visualisation of the vasculature in these pathologies requires intravenous administration of high molecular weight blood-pool contrast agents, similar to CT angiography. This imaging modality has also been used to identify the presence of thrombus in a murine model of inferior vena cava (IVC) thrombosis [3], however, a detailed characterisation for the longitudinal assessment of thrombus resolution has yet to be reported. The aim of the present study was to assess the applicability of contrast-enhanced microCT for longitudinal measurement of thrombus resolution.

## 2 Materials and methods

### 2.1 St. Thomas' model of venous thrombosis

Thrombi were induced in the inferior vena cava (IVC) of 8–10 week-old male Balb/c mice as previously described [12]. Briefly, a midline laparotomy was carried out under general anaesthesia and sharp dissection used to separate the IVC from the aorta inferior to the left renal vein. A piece of 4–0 mersilk suture (Ethicon, USA) was slung around the IVC and tied onto a piece of 5–0 prolene suture (Ethicon, USA) laid along the vessel which was then removed to generate a 90% stenosis of the inferior vena cava. A neurovascular clip (Fine Scientific Tools, Germany) was applied to the infra-renal segment of the IVC to induce endothelial dysfunction. All procedures were carried out in accordance with the UK Animals (Scientific Procedures) Act, 1986.

### 2.2 MicroCT

Mice were anaesthetised under 3% isoflurane at an oxygen flow rate of 1 l/min. Aurovist 15 nm nanogold (nAu) contrast agent (Nanoprobes, USA, 200 mg/ml nAu) was diluted with saline to a concentration of 50 mg/ml nAu. A 200 μl bolus of contrast agent was administered by intravenous injection into the tail vein giving a nAu dose of 10 mg/mouse. Contrast was allowed to circulate for 5 min prior to commencement of the scan.

A nanoScan PET/CT 8W (Mediso Ltd., Hungary) was used to image the thrombosed IVC. The mouse was placed on the scanner bed in the prone position and a pressure transducer placed to measure respiration rate. An initial scout view was obtained to allow specification of the anatomical limits of the high-resolution scan. A high-resolution scan (maximum zoom, 360 projections, pitch 1, 45 kVP, 1000 ms exposure) was completed taking approximately 20 min.

### 2.3 Image reconstruction

Scans were reconstructed using VivoQuant software (v1.22, Invicro, USA) with a voxel size of 65 μm. Reconstructed scans were segmented and analysed using ITKsnap software (v2.4, Open Source) [13]. Image contrast was adjusted linearly to provide optimal parameters for subsequent analysis. Thrombi were reconstructed in 3D using a semi-automatic volumetric bubble propagation system with a balloon expansion force of 1.00 and a curvature term of 0.20. Manual corrections to the segmentation were made using the free-hand tool. When the segmentation was complete a 3D rendered mesh of the thrombus was generated and thrombus volume recorded. A representative example of segmentation of the thrombus from surrounding tissue has been provided in supplementary material (Fig. S1).

### 2.4 Histology

The thrombosed IVC was excised from the site of stenosis to the iliac bifurcation at 1, 7 and 14 days post-induction. Samples were fixed in 10% formol saline for 18 h and processed in a Leica TP1020 tissue processor (Leica, Germany). 5 μm paraffin sections were cut at 500 μm intervals throughout the length of the thrombus. Haematoxylin and eosin (H&E) staining was used to visualise the thrombus within the IVC as previously described [12]. Perls's staining was used to identify intracellular iron deposits in thrombus sections by incubation with a 2%(*w*/*v*) potassium ferrocyanide in 2%(*v*/*v*) hydrochloric acid solution for 20 min, followed by a distilled water wash and counterstaining for 5 min with Nuclear Fast Red (Vector Laboratories, UK). All sections were dehydrated and mounted in DPX medium (Sigma, USA).

### 2.5 Immunohistochemistry

Macrophages and neovascular channels were localised in the thrombus by immunohistochemical localisation of Mac2 (galectin3) and CD31 expression respectively, as previously described [12]. In brief, sections were dewaxed in xylene and rehydrated through graded alcohols. Heat-mediated antigen retrieval was performed in citrate buffer (pH 6). Sections were blocked in serum-free protein solution (Dako, UK) prior to incubation with either rat anti-mouse Mac2 (Biolegend, UK) or CD31 (Abcam, UK) for 1 h at room temperature. Sections were incubated with appropriate biotinylated secondary antibodies for 45 min at room temperature. Signal amplification was achieved by incubation with extravidin–peroxidase conjugates (Sigma, USA) for 1 h at room temperature (Mac2) or with X-cell Plus HRP polymer (Menarini, UK) for 30 min at room temperature (CD31). Specific antigen–antibody conjugates were visualised with either NovaRed or Vector SG (Vector Laboratories, UK) chromagen substrates. Sections were counterstained with Mayer's haemalum (ClinTech, UK) or Nuclear Fast Red (Vector Laboratories, UK), dehydrated and mounted in DPX medium.

### 2.6 Image capture and analysis

H&E stained thrombus sections were imaged using a QICAM camera (Q Imaging, Canada) attached to a brightfield microscope (Leica DMRB, Leica, Germany). Thrombus cross-sectional areas were calculated by analysis of micrographs using Image Pro Plus software (MediaCybernetics, UK) as previously described [12].

### 2.7 Statistics

Differences in thrombus volume over time, measured by histology and microCT, were assessed by appropriate paired and unpaired one-way analysis of variance (ANOVA) with differences between individual time-points determined using Bonferroni's post-hoc tests. Endpoint analysis of thrombus volume, as determined by both microCT and histology, was assessed by Wilcoxon's test. Relationships between measurements of thrombus volume were examined by intra-class correlation with data represented graphically using linear regression and Bland–Altman plots. Spearman's correlation was used to investigate the relationship between contrast extravasation and thrombus organisation. Data is represented either as individual data points or mean ± standard error.

## 3 Results

**Fig. 1:**
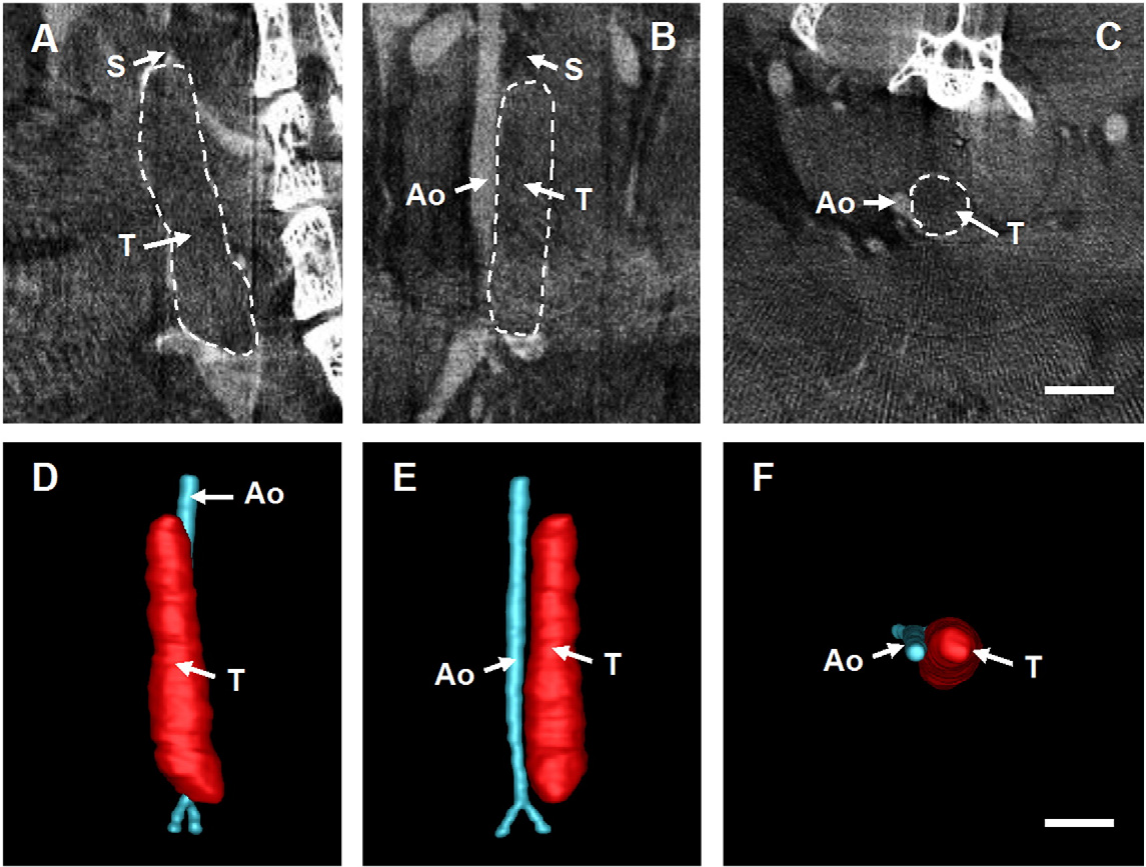
Imaging of IVC thrombi by contrast-enhanced microCT. A representative day 1 thrombus imaged by contrast-enhanced microCT shown in transverse (A), sagittal (B) and coronal (C) planes (scale bar 2 mm). Segmentation of the scan allow for reconstruction of 3D volume renders (D–F). Images have been annotated with the position of the aorta (Ao), the site of stenosis (S) and the thrombus (T).

IVC resident thrombus was visualised *in situ* by contrast-enhanced microCT. Thrombus presented as a hypo-perfused region distal to the site of stenosis (Fig. 1A–C). The presence of a perfusion defect in the IVC lumen enabled segmentation of the thrombus from surrounding tissue and subsequent reconstruction of 3D volume renders of the thrombus and surrounding vascular features (Fig. 1D–F).

**Fig. 2:**
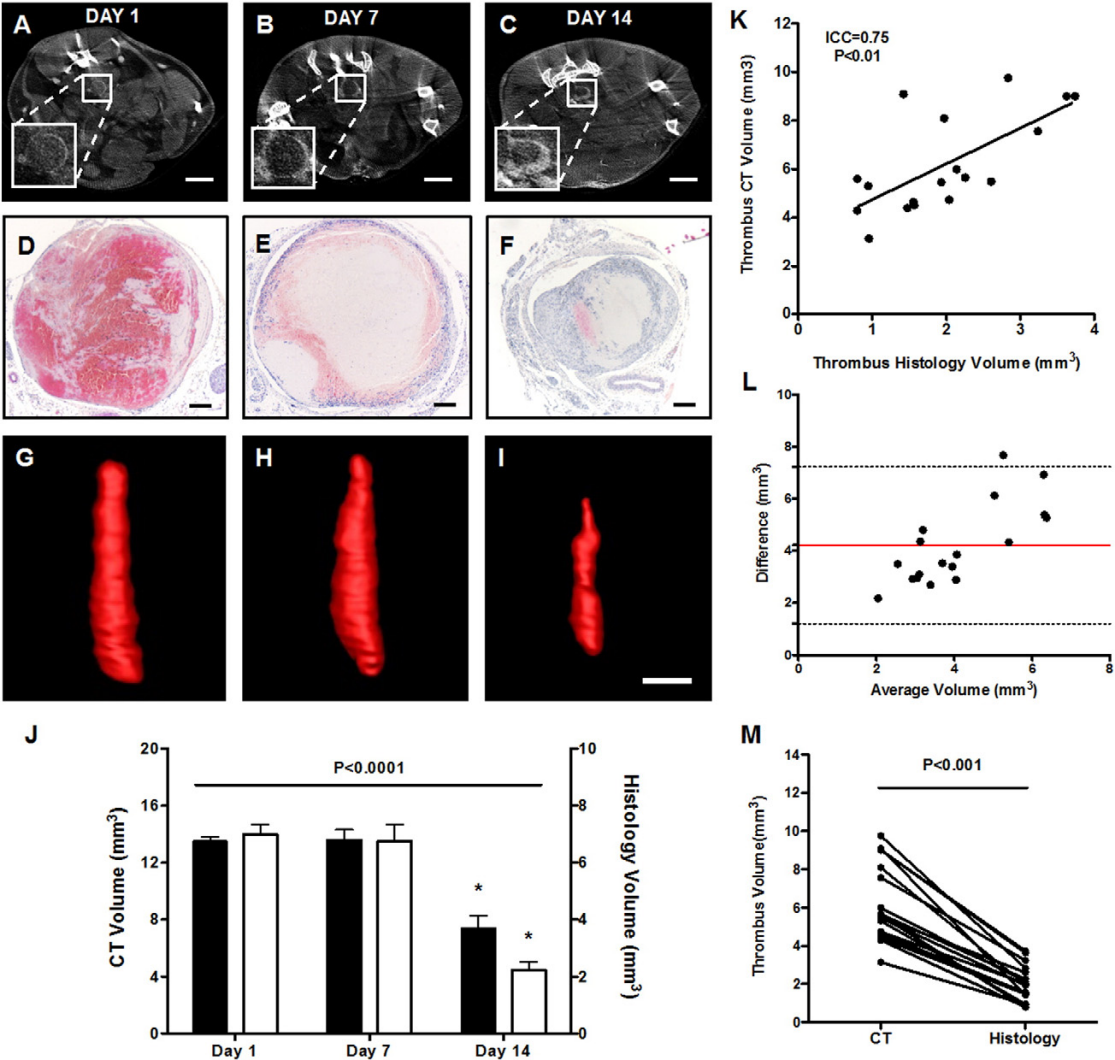
Visualisation of the thrombus by histology and microCT. Thrombus was imaged at days 1, 7 and 14 post-induction by contrast-enhanced microCT (n = 6 per timepoint). (A–C) Representative transverse slices demonstrate the presence of thrombus in the IVC lumen (scale bar 2 mm), (D–F) corresponding with time-matched H&E stained thrombus micrographs (scale bar 200 μm). (J) Thrombus volume of time-matched samples determined by microCT (black bars, n = 6 per timepoint) and histology (white bars, n = 5 per timepoint) demonstrate similar temporal changes in thrombus volume (one-way ANOVA, P < 0.0001). (K) Measurements of thrombus volume by microCT and histology were positively correlated (ICC = 0.75, P < 0.01), however, (L) corresponding Bland–Altman demonstrated a marked bias in absolute thrombus volume (M) more readily appreciated by paired analysis (Wilcoxon test, P < 0.001, n = 18 mice).

At day 1 post-induction the thrombus was largely occlusive with small regions of perfused lumen evident (Fig. 2A). At day 7 post-induction a small amount of luminal recanalisation was apparent (Fig. 2B) although the majority of thrombi remained occlusive at some point along the length. By day 14 post-induction marked recanalisation of the vein lumen was noted (Fig. 2C) indicative of extensive remodelling of the thrombus. This pattern of vein lumen recanalisation is consistent with histological analyses of time-point-matched samples (Fig. 2D–F). Generation of 3D volume renders of the thrombus revealed that whilst the acute day 1 thrombus appeared largely cylindrical, the resolving thrombus at day 7 and particularly at day 14 was heterogeneous with respect to cross-sectional area (Fig. 2G–I). This matches with histological observations of thrombus cross-sectional area (Fig. S2, Supplementary material).

Similar temporal changes in thrombus resolution were observed by both contrast-enhanced microCT and histology with thrombus volume significantly reduced at day 14 post-induction compared to days 1 and 7 (P < 0.0001, Fig. 2J). Paired analysis of thrombus volume at day 14 demonstrated a strong positive correlation between microCT and histological measurements (ICC = 0.75, P < 0.01, Fig. 2K). Differences in absolute thrombus volume were, however, apparent between the two techniques. Bland–Altman analysis and comparison of paired measurements of day 14 thrombi revealed a markedly higher thrombus volumes (~ 4 mm^3^) measured by microCT (Fig. 2L), compared with histological analysis, which consistently and significantly underestimate thrombus volume (P < 0.001, Fig. 2M).

**Fig. 3:**
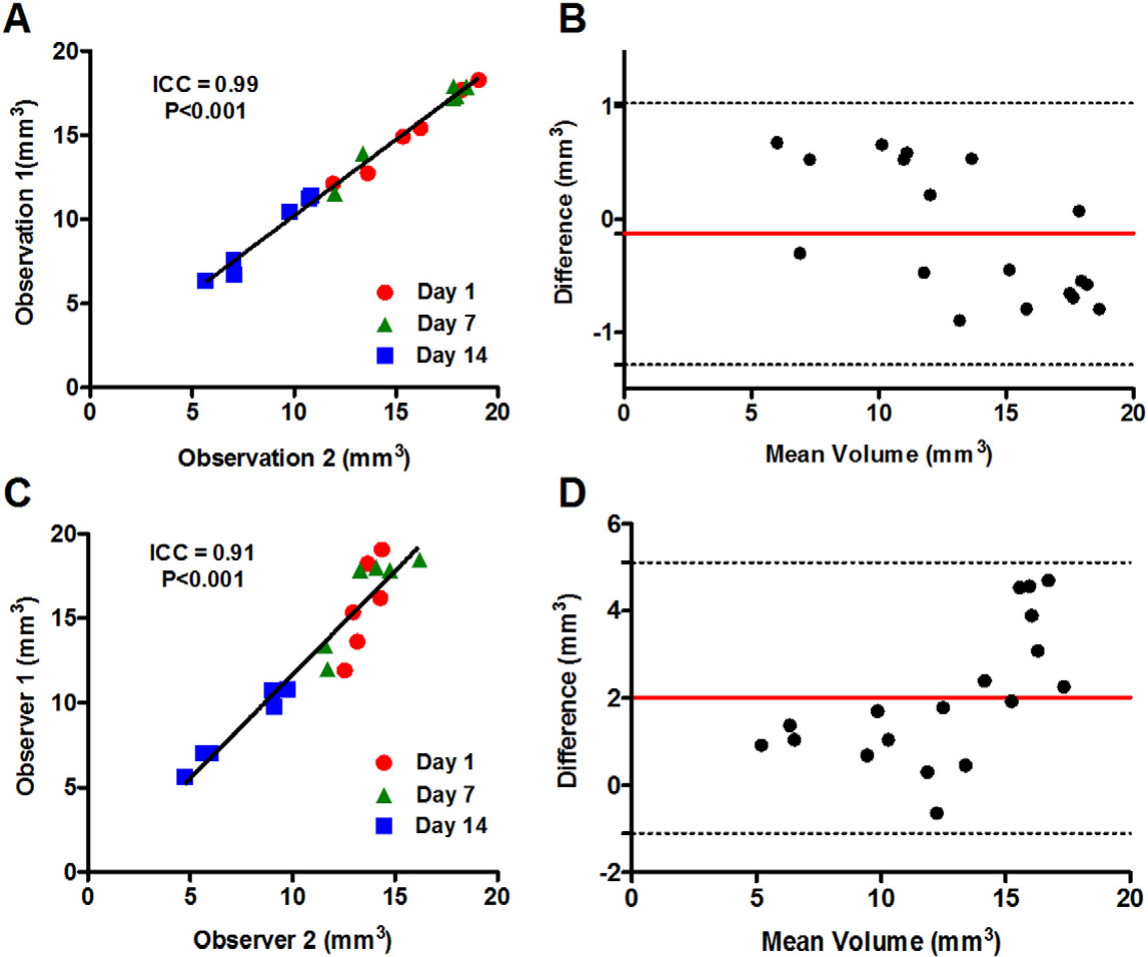
Intra- and inter-observer variability of thrombus volume measurements by microCT. The reliability and reproducibility of thrombus volume measurements were assessed by both intra- and inter-observer analyses of thrombi imaged at days 1, 7 and 14 post-induction (n = 6 mice per timepoint). (A) Intra-observer measurements correlated well (ICC = 0.99, P < 0.0001) (B) with no apparent bias between datasets (Bland–Altman). (C) Inter-observer measurements were also well correlated (ICC = 0.91, P < 0.0001), however, (D) a marked bias in mean thrombus volume was apparent (Bland–Altman).

Variability in measurement of thrombus volume by microCT was compared after either multiple measurements by a single observer (intra-observer) or after measurements by multiple observers (inter-observer). Intra-observer measurements of thrombus volume showed a strong positive correlation (ICC = 0.99, P < 0.0001, Fig. 3A), with the corresponding Bland–Altman plot demonstrating negligible bias (Fig. 3B). Inter-observer measurements also showed a strong positive correlation (ICC = 0.91, P < 0.0001, Fig. 3C), although Bland–Altman analysis showed a bias between measurements (~ 2 mm^3^) that appeared to increase with size of the thrombus (Fig. 3D).

**Fig. 4:**
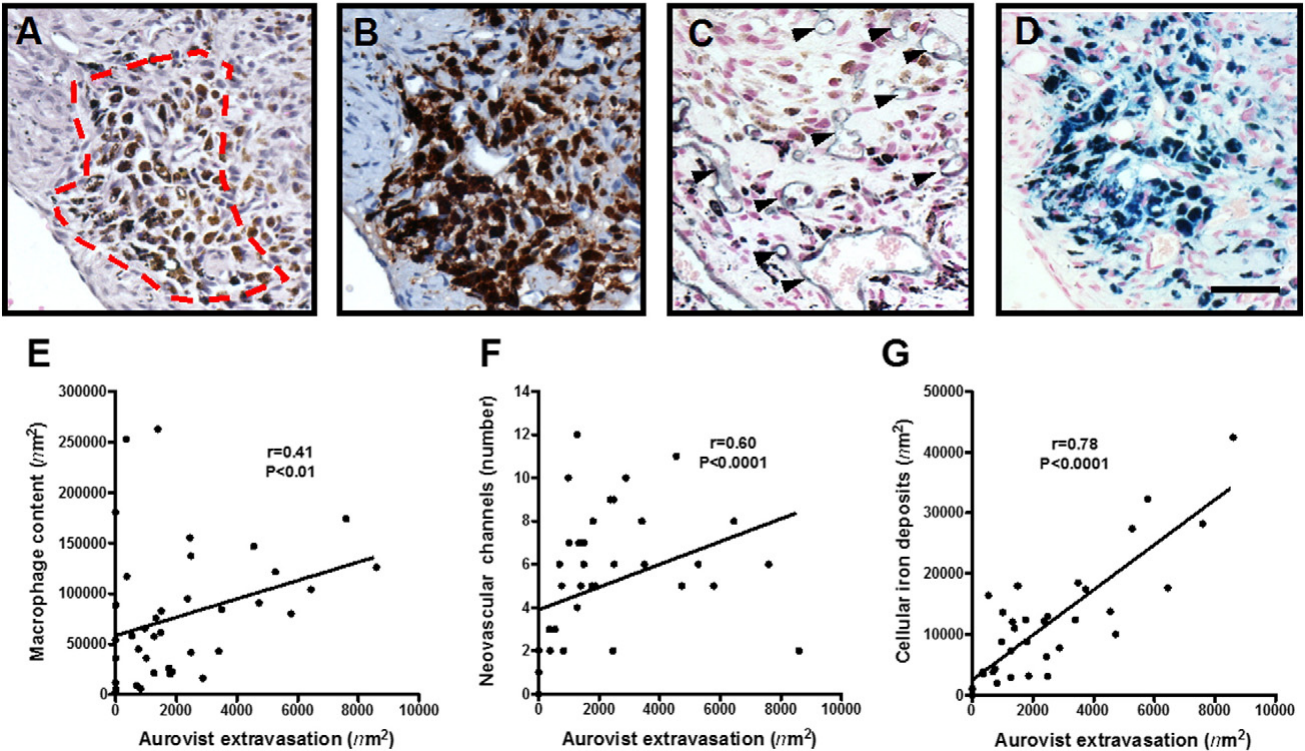
Aurovist contrast agent extravasation. Aurovist contrast extravasation (dark staining) was observed in (A) H&E stained sections of day 14 thrombi. Contrast extravasation occurred in highly organised regions of the thrombus rich in (B) Mac-2 positive macrophages and (C) CD31 positive neovascular channels (arrow heads) and (D) cellular iron deposits (scale bar 40 μm). The area of thrombus containing Aurovist correlated with (E) macrophage content (r = 0.41, P < 0.01), (F) neovascularisation (r = 0.60, P < 0.0001) and (G) iron deposits (r = 0.78, P < 0.0001), Spearman's rank n = 38 sections per group.

Histological examination of thrombi imaged longitudinally by microCT and harvested at 14 days post-induction, revealed dense black regions of extravasated contrast-agent in the thrombus periphery in H&E stained sections (Fig. 4A). Contrast-agent extravasation correlated with thrombus Mac2 macrophage content (r = 0.41, P < 0.01, Fig. 4B and E), the extent of CD31 positive neovascular channels (r = 0.60, P < 0.0001, Fig. 4C and F) and iron deposits localised by Perl's staining (r = 0.78, P < 0.0001, Fig. 4D and G).

## 4 Discussion

Contrast-enhanced microCT provides a rapid, reliable and sensitive imaging modality for the longitudinal measurement of thrombus volume in an established model of thrombosis [1]. Contrast-enhanced microCT was successful in delineating the thrombus from the surrounding tissue in all scans with a typical scan time of approximately 20 min. The imaging protocol used was well tolerated with no adverse effects associated with either repeated contrast injection or the microCT protocol used.

Contrast-enhanced microCT identifies thrombus as a perfusion defect within the vessel lumen. Consistent with previous studies, acute thrombi, such as at day 1 post-induction, were largely occlusive in nature [5]. Contrast agent was observed in proximal and distal segments of acute thrombi at day 1 post-induction. This signal may be attributed to small regions of perfused IVC lumen or alternatively extravasation of contrast agent into the vessel wall. The limited amount of contrast-enhancement between the thrombus and the vein wall at acute time-points made accurate segmentation challenging. This was overcome by registration with anatomical structures surrounding the thrombus including: the site of stenosis, aorta and distal segment of the IVC.

The dynamics of thrombus resolution in this study were broadly consistent with previous reports using the same model [14]. Comparison of measurements taken by microCT and histology show similar temporal changes in thrombus volume during resolution. Both measures demonstrated that thrombus volume was similar between days 1 and 7 post-induction, but significantly reduced between days 7 and 14. Direct comparison of paired microCT and histology measurements at day 14 revealed a strong positive correlation, albeit thrombus volumes determined by histology were consistently smaller than those values obtained by microCT. A possible reason for the observed differences in thrombus size measured by the two techniques is the occurrence of tissue shrinkage during processing for histological analysis [15].

Intra- and inter-observer variability were assessed in order to examine the reliability of image analysis of the microCT scans. The strong positive correlation between intra-observer measurements indicated that the thrombus could be segmented in a reproducible manner by an individual observer. However, although inter-observer measurements showed a strong positive correlation, there may be bias in the segmentation of the thrombus by different observers. To limit the effect of observer dependent bias it may be advantageous for individual data sets to be analysed by a single observer in a blinded manner.

The nAu contrast agent Aurovist used in this study provides a high level of contrast enhancement compared to alternative agents such as iomeprol and ExiTron nano12000 [16]. This approach allows for relatively low volumes of contrast agent to be administered. Good blood pool retention of Aurovist has been reported, which allows for injection of a single bolus of contrast-agent rather than constant infusion used with traditional ionidated agents [16]. Aurovist is cleared by the kidneys, in a manner similar to iodinated agents, but this is not associated with the same degree of nephrotoxicity [17]. These characteristics make Aurovist a suitable contrast agent for longitudinal imaging of the vasculature.

It was not unexpected to find Aurovist extravasating into the thrombus given the size of these particles (~ 15 nm). Histological analysis of thrombi confirmed the presence of the Aurovist in highly organised regions of the thrombus with extensive neovascularisation and rich in macrophage/haemosiderin-containing cells. This could have been as a result of vascular hyperpermeability similar to that observed in tumour neovessels [18]. Subsequent uptake of Aurovist particles by thrombus-resident macrophages may have occurred, as demonstrated by a number of *in vitro* studies [19], [20]. Extravasation of contrast-agent is most likely to have taken place after day 7, the time-point at which neovascular channel formation and macrophage ingress are first observed [12].

With respect to clinical applicability, contrast-enhanced CT is not commonly used in the diagnosis of DVT. Instead, non-contrast duplex ultrasonography is currently considered the gold-standard imaging technique for diagnosis [21]. The development of clinical 3D ultrasound devices has enabled longitudinal, quantitative measurements of thrombus volume and represents a complementary technique for assessment of thrombus resolution in man [22].

To conclude, the data provided in this study supports the utility of contrast-enhanced microCT in longitudinal measurement of thrombus in experimental models. This imaging modality provides powerful, paired *in vivo* data of thrombus resolution, potentially reducing the number of animals required for experimentation.

## Funding

This study was funded by a non-clinical PhD studentship from the British Heart Foundation to SG (FS/10/51/28677). The PET/CT scanning equipment at King's College London was funded by an equipment grant from the Wellcome Trust (WT 084052/Z/07/Z). The funders had no role in study design, data collection, data analysis, decision to publish, or preparation of the manuscript.

## References

[1] Singh I., Burnand K.G., Collins M., Luttun A., Collen D., Boelhouwer B. (2003). Failure of thrombus to resolve in urokinase-type plasminogen activator gene-knockout mice: rescue by normal bone marrow-derived cells. Circulation.

[2] Nosaka M., Ishida Y., Kimura A., Kuninaka Y., Inui M., Mukaida N. (2011). Absence of IFN-gamma accelerates thrombus resolution through enhanced MMP-9 and VEGF expression in mice. J. Clin. Invest..

[3] von Bruhl M.L., Stark K., Steinhart A., Chandraratne S., Konrad I., Lorenz M. (2012). Monocytes, neutrophils, and platelets cooperate to initiate and propagate venous thrombosis in mice in vivo. J. Exp. Med..

[4] Phinikaridou A., Andia M.E., Saha P., Modarai B., Smith A., Botnar R.M. (2013). In vivo magnetization transfer and diffusion-weighted magnetic resonance imaging detects thrombus composition in a mouse model of deep vein thrombosis. Circ. Cardiovasc. Imaging.

[5] Saha P., Andia M.E., Modarai B., Blume U., Humphries J., Patel A.S. (2013). Magnetic resonance T1 relaxation time of venous thrombus is determined by iron processing and predicts susceptibility to lysis. Circulation.

[6] Aghourian M.N., Lemarie C.A., Blostein M.D. (2012). In vivo monitoring of venous thrombosis in mice. J. Thromb. Haemost..

[7] Guenther F., Herr N., Mauler M., Witsch T., Roming F., Hein L. (2013). Contrast ultrasound for the quantification of deep vein thrombosis in living mice: effects of enoxaparin and P2Y12 receptor inhibition. J. Thromb. Haemost..

[8] Ritman E.L. (2011). Current status of developments and applications of micro-CT. Annu. Rev. Biomed. Eng..

[9] Takeda Y., Costa S., Delamarre E., Roncal C., Leite de Oliveira R., Squadrito M.L. (2011). Macrophage skewing by Phd2 haplodeficiency prevents ischaemia by inducing arteriogenesis. Nature.

[10] Kaijzel E.L., van Heijningen P.M., Wielopolski P.A., Vermeij M., Koning G.A., van Cappellen W.A. (2010). Multimodality imaging reveals a gradual increase in matrix metalloproteinase activity at aneurysmal lesions in live fibulin-4 mice. Circ. Cardiovasc. Imaging..

[11] Nahrendorf M., Badea C., Hedlund L.W., Figueiredo J.L., Sosnovik D.E., Johnson G.A. (2007). High-resolution imaging of murine myocardial infarction with delayed-enhancement cine micro-CT. Am. J. Heart Circ. Physiol..

[12] Evans C.E., Grover S.P., Humphries J., Saha P., Patel A.P., Patel A.S. (2014). Antiangiogenic therapy inhibits venous thrombus resolution. Arterioscler. Thromb. Vasc. Biol..

[13] Yushkevich P.A., Piven J., Hazlett H.C., Smith R.G., Ho S., Gee J.C. (2006). User-guided 3D active contour segmentation of anatomical structures: significantly improved efficiency and reliability. NeuroImage.

[14] Frey M.K., Alias S., Winter M.P., Redwan B., Stubiger G., Panzenboeck A. (2014). Splenectomy is modifying the vascular remodeling of thrombosis. J. Am. Heart Assoc..

[15] Fox C.H., Johnson F.B., Whiting J., Roller P.P. (1985). Formaldehyde fixation. J. Histochem. Cytochem..

[16] Nebuloni L., Kuhn G.A., Muller R. (2013). A comparative analysis of water-soluble and blood-pool contrast agents for in vivo vascular imaging with micro-CT. Acad. Radiol..

[17] Hainfeld J.F., Slatkin D.N., Focella T.M., Smilowitz H.M. (2006). Gold nanoparticles: a new X-ray contrast agent. Br. J. Radiol..

[18] Nagy J.A., Chang S.H., Shih S.C., Dvorak A.M., Dvorak H.F. (2010). Heterogeneity of the tumor vasculature. Semin. Thromb. Haemost..

[19] Krpetic Z., Porta F., Caneva E., Dal Santo V., Scari G. (2010). Phagocytosis of biocompatible gold nanoparticles. Langmuir.

[20] Liu X., Huang N., Li H., Jin Q., Ji J. (2013). Surface and size effects on cell interaction of gold nanoparticles with both phagocytic and nonphagocytic cells. Langmuir.

[21] Zierler B.K. (2004). Ultrasonography and diagnosis of venous thromboembolism. Circulation.

[22] Zhao L.P., S.J., Kampmann M., Sorkin J.D., Caldwell K., Braganza M., McEvoy S., Lal B.K. (2014). Measurement of thrombus resolution using three-dimensional ultrasound assessment of deep vein thrombosis volume. J. Vasc. Surg. Ven. Lymphat. Disord..

