# Supplementary material for: Quantification of experimental venous thrombus resolution by longitudinal nanogold-enhanced micro-computed tomography
Source: Thromb Res. 2015 Dec;136(6):1285–90. doi: 10.1016/j.thromres.2015.10.006 (PMC4697135; doi:10.1016/j.thromres.2015.10.006)
Supplement: Supplementary file 1 — Supplementary figures. [file mmc1.docx]

**Supplementary material**

**Figure S1: Contrast-enhanced microCT thrombus segmentation**

Using ITK-SNAP software (A) a small region of interest was selected (red box). (B) The scan was processed to restrict “bubble propagation” to regions of hypo-intensity (white area). (C) Small “bubbles” were placed along the length of the thrombus and (D) propagated until the thrombosed region was filled. (E) The segmentation was manually refined until all of the thrombus was selected before (F) a 3D volume render was generated from which a measurement of thrombus volume was taken.

**Figure S2: Thrombus cross-sectional area**

(A) Representative H&E stained thrombus sections at days 1, 7 and 14 post-induction (scale bar = 200μm). (B) Thrombus cross-sectional area (n=32-37 / time-point) at days 1, 7 and 14 post-induction was found to decrease significantly over time (P<0.0001, Kruskal-Wallis), data represented by box-and-whisker plots (median, interquartile range, range).

**Figure S1**

**Figure S2**
